# Prevalence of Bovine Mastitis and Its Associated Risk Factors among Dairy Cows in Ethiopia during 2005–2022: A Systematic Review and Meta-Analysis

**DOI:** 10.1155/2022/7775197

**Published:** 2022-09-17

**Authors:** Abayeneh Girma, Dessalew Tamir

**Affiliations:** ^1^Department of Biology, College of Natural and Computational Science, Mekdela Amba University, P.O. Box 32, Tuluawlia, Ethiopia; ^2^Department of Veterinary Science, College of Agriculture and Environmental Sciences, Debre Tabor University, P.O. Box 272, Debre Tabor, Ethiopia

## Abstract

Bovine mastitis remains a major prevalent disease in cattle and places a significant economic burden on the global dairy industry. The goal of this systematic review and meta-analysis was to examine the overall prevalence of mastitis and its associated risk factors among dairy cows. Scientific articles written in English were recovered from PubMed, ScienceDirect, Web of Science, Google Scholar, Cochrane Library, and other sources from Google Engine and University Library Databases. “Prevalence,” “bovine mastitis,” “clinical mastitis,” “subclinical mastitis,” “associated factors,” “dairy cows,” and “Ethiopia” were search terms used for this study. For critical appraisal, PRISMA 2009 was applied. Heterogeneity and publication bias were evaluated using Cochran's *Q*, inverse variance (*I*^2^), and funnel plot asymmetry tests. A random-effects model was used to calculate the pooled burden of mastitis and its associated factors among dairy cows, along with the parallel odds ratio (OR) and 95% confidence interval (CI). A total of 6438 dairy cows were included in the 17 eligible studies for this meta-analysis. The overall pooled prevalence of mastitis among dairy cows in Ethiopia was 43.60% (95% CI: 34.71, 52.49), of which 12.59% (95% CI: 7.18, 18.00) and 32.21% (95% CI: 24.68, 39.74) were clinical and subclinical cases, respectively. Of the regions, the highest and lowest pooled prevalence estimates of mastitis among dairy cows were 49.90% (95% CI: 31.77, 68.03) and 25.09% (95% CI: 3.86, 46.32) in the Oromia and Amhara regions, respectively. The highest pooled prevalence estimate in the study period was recorded between 2017 and 2022, with a pooled prevalence estimate of 46.83% (95% CI: 35.68, 57.97), followed by the study period from 2005 to 2016, with a pooled prevalence estimate of 39.97% (95% CI: 25.50, 54.44). Gram-positive bacteria (84.70%) were the most prevalent mastitis-causing agents compared with Gram-negative bacteria (15.30%). Breed (AOR: 2.17, 95% CI: 1.44, 2.90), lactation stage (AOR: 1.59, 95% CI: 1.04, 2.15), parity (AOR: 3.31, 95% CI: 1.69, 4.94), history of mastitis (AOR: 3.56, 95% CI: 2.40, 4.71), floor type (AOR: 1.59, 95% CI: −0.16, 3.34), and teat injury (AOR: 6.98, 95% CI: 0.33, 13.64) were factors significantly associated with mastitis among dairy cows in Ethiopia. Early diagnosis and proper medication, as well as implementing appropriate prevention and control measures, are necessary for the management of mastitis in dairy cows.

## 1. Introduction

Ethiopia's economy is mostly reliant on agriculture, with crop and livestock production in the highlands and primarily livestock production in the lowlands. Livestock is a vital aspect of Ethiopia's agricultural production system and is a key national resource. In Ethiopia, there are an estimated 57.83 million cattle (55.38% female and 44.62% male cattle, of which 11.66 were dairy cows), 28.89 million sheep, 29.70 million goats, 10 million equines, 1.2 million camels, and more than 60.51 million chickens, and tremendous bee and fishery resources [1, 2]. This makes it home to the largest livestock population in any African country. In Ethiopia, 98.20% of the total cattle is local breeds, and the rest are cross-breeds and exotics, which represent approximately 1.62% and 0.18%, respectively. The livestock subsector is critical to the Ethiopian economy as a source of food, income, services, and foreign exchange, accounting for 16.5% of total GDP and 45% of agricultural GDP, respectively [3–5]. In addition, it contributes 12 to 15% of total export revenues, placing it second in importance [6].

In Ethiopia, dairy farming is mostly managed through an extensive system that includes smallholder farmers in rural areas. Currently, semi-intensive and intensive dairy production systems are gaining popularity among farmers with good market access. However, important impediments to dairy production include the low genetic potential of indigenous cattle breeds, diseases, insufficient feed and water, and slow progress in dairy development technologies. With the introduction of alien breeds into the country for superior genetics and milk production, disease is becoming a major concern. Mastitis is one of the diseases that are known to be prevalent in various dairy production systems around the country, resulting significant economic losses. Mastitis impacts the quality and quantity of milk by causing physical, chemical, and bacterial changes in the milk and pathological changes in the glandular tissue of the udder. It has several negative consequences, including a lower milk yield, increases culling rates and treatment expenses, and accelerates the possibility of death from severe diseases [3]. In addition, some udder infections, such as *Staphylococcus aureus*, impact food safety by producing toxins that cause food poisoning [7].

Mastitis is an inflammation of the parenchyma of mammary gland produced by infectious agents that infiltrate the udder, multiply, and produce toxins. To date, more than 140 potentially pathogenic organisms have been identified that cause cow mastitis. The disease is divided into four categories based on the organism involved: bacterial, mycotic/fungal/algal, Mycoplasmal, and Nocardial mastitis. The viruses are of minor clinical importance. Mastitis is a multifactorial disease involving microbes, the host, and the environment. According to epidemiology, mastitis is classified as contagious or environmental. Contagious mastitis is an intramammary infection (IMI) transmitted from a cow with an infected udder to a healthy cow. A wide range of contagious pathogens are found in cows' udders, including *Staphylococcus aureus*, *Streptococcus agalactiae*, *Mycoplasma spp.*, and *Corynebacterium bovis* [8]. On the contrary, environmental mastitis occurs when infections are caused by pathogens whose primary reservoir is the environment in which the cow lives. Most infections caused by environmental pathogens are clinical and short-lasting, including *Escherichia coli*, *Klebsiella* species, *Streptococcus dysgalactiae*, and *Streptococcus uberis* [9–12].

On the other hand, mastitis is also dividedinto clinical (symptomatic mastitis=mastitis with visible symptoms) and subclinical (asymptomatic mastitis=mastitis without visible symptoms) forms. Clinical mastitis is characterized by the presence of indications of inflammation in the mammary glands, such as swelling, heat, pain, and edema, as well as changes in the milk, such as flakes and clots [10–12]. Clinical mastitis further poses a hazard to animal welfare since it causes pain, a rise in mean rectal temperature, a rise in heart rate, and a rise in respiration rate. Comparatively, subclinical mastitis is an inflammation of the mammary gland that occurs without obvious signs and can progress to clinical mastitis or vice versa [11-15]. This type of mastitis results in a nonevidenced decrease in milk production, as well as changes in milk quality and content. The loss of quarter(s) or teat(s) might occur as a result of severe or chronic inflammation [16]. Cows with blind quarters produce less and are more likely to be killed early than their healthy counterparts [17]. Furthermore, the removal of milk from lactating animals with mastitis causes significant food losses, which in turn results in nutritional insufficiency in children and nursing mothers, ultimately resulting in diseases of nutritional deficiency [13, 14]. With regard to prevalence, several studies were conducted in small- and large-scale dairy farms of Ethiopia and found a prevalence range of 2.7–21.0% for clinical mastitis and 33.3–68.1% for subclinical mastitis cases [18, 19]. A variety of reports have shown that different breeds and regions of Ethiopia have different levels of prevalence of mastitis. Additionally, these studies showed that a variety of factors influence bovine mastitis at the farm and individual animal levels [3-7, 20-22].

In Ethiopia, mastitis not only has an impact on animal health and well-being but can also have a significant impact on dairy profitability, financial loss, and public health. Ethiopia generates 3.2 billion liters per day from 10 million milking cows, averaging 1.54 liters per cow per day over a 180-day lactation cycle. Mastitis is responsible for 78% of the overall loss in milk production of Ethiopia. In addition to reproductive diseases, approximately 140 to 200 USD/cow/year is a key reason for Ethiopia's economic failure. Mastitis causes an economic loss of 58 and 78.65 USD per cow and per lactation in Addis Ababa's urban and peri-urban areas, respectively. Losses were largest in large-scale farms (13%) and lowest in small-scale farms (3.7%), with an overall financial loss per cow each lactation of 78.65 USD and losses in large farms of 150.35 USD [23].

Identification (screening tests, physical and bacteriological examinations) of the types of organisms that cause mastitis in dairy cows, as well as the selection of an effective antimicrobial agent against the organism in question, is critical to the successful care of animals and public health. The rise of resistant bacterial strains in cows and milk continues to represent a problem in terms of treating and controlling the transmission of disease. Furthermore, the indiscriminate use of antibiotics frequently leads to an increase in dairy pathogen resistance to the most commonly used antimicrobial medications, especially in cows. Although mastitis rarely causes complications, it can have serious consequences in terms of morbidity and mortality. In addition, the isolates were found to have a significant level of resistance to routinely used antibiotics, leaving clinicians with a limited number of options for treating mastitis-causing bacterial pathogens.

Bovine mastitis is one of the animal and public health problems of Ethiopia, with varying levels of prevalence throughout the country. However, in Ethiopia, the prevalence of mastitis among dairy cows and its predisposing factors are not collected, well-organized, or recorded as a systematic review and meta-analysis. As a result, the purpose of this study was to provide evidence on the overall prevalence and risk factors for mastitis among dairy cows using previously conducted research articles found in different regions of Ethiopia. Furthermore, the results obtained in the current investigation could contribute significantly to policymakers, development planners, and animal health practitioners.

## 2. Methods

### 2.1. Profile of the Country

Ethiopia measures 1,104,300 square kilometers and is located in the Horn of Africa. The total land area is 1,000,000 square kilometers (386,102 square miles). Ethiopia is bordered in the north by Eritrea, in the east by Djibouti and Somalia, in the west by Sudan and South Sudan, and in the south by Kenya. According to Worldometer's elaboration of the most recent United Nations data, Ethiopia's current population was 113,881,451 in 2020, which is comparable to 1.47%. Furthermore, according to the aforementioned report, approximately 21.3% of the population (24,463,423) will live in urban areas by 2020 [24].

### 2.2. Search Strategy

**Figure 1:**
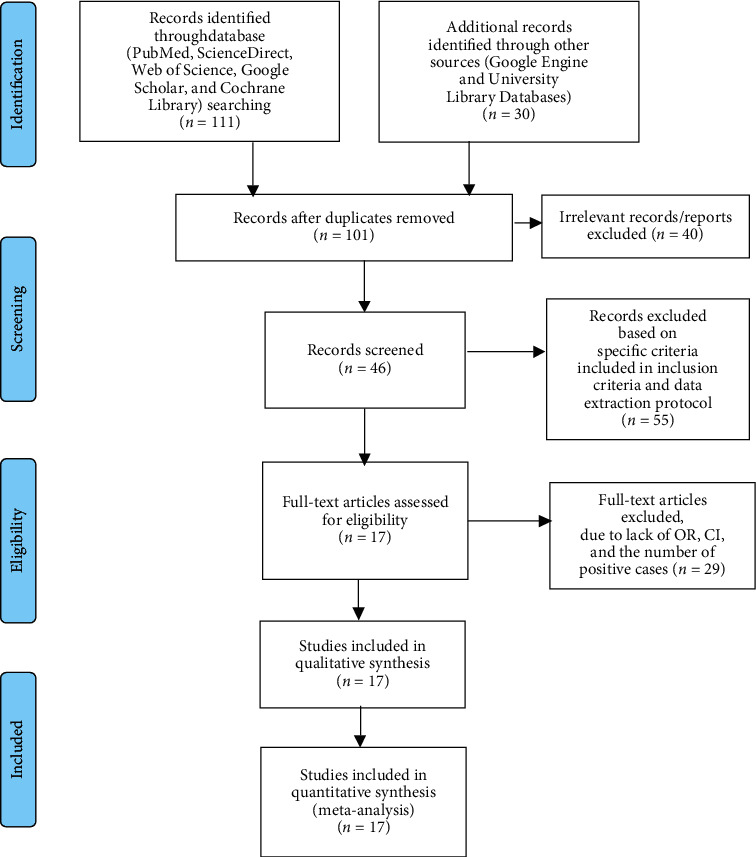
PRISMA 2009 flow diagram of eligible studies.

This systematic review and meta-analysis were performed according to the Preferred Reporting Items for Systematic Review and Meta-Analysis guidelines [25]. An extensive search was conducted in international databases (PubMed, ScienceDirect, Web of Science, Google Scholar, and Cochrane Library) and other sources (Google Engine and University Library Databases). Articles were searched using MeSH key terms and phrases in combination or separate using “AND”/OR” such as “prevalence,” “bovine mastitis,” “clinical mastitis,” “subclinical mastitis,” “associated factors,” “dairy cows,” and “Ethiopia.” The study was carried out from January to June 2022. The search process was presented following PRISMA flow chart 2009 guidelines that clearly indicate the studies included and excluded with reasons of exclusion (Figure 1).

### 2.3. Criteria for Inclusion and Exclusion of Studies

Articles collected through the searches were evaluated for inclusion in the meta-analysis based on the following criteria: (i) Ethiopian studies on the prevalence of bovine mastitis and its risk factors in dairy cows with at least 100 observations; (ii) only category of animal studies and reported in English with clearly stated sample sizes, number of positive samples, and study locations; (iii) cross-sectional studies; (iv) journals studied from 2005 to 2022; and (v) only bacterial etiological agents of bovine mastitis; (vi) articles published and unpublished (1 from Bahir Dar University library databases), which are available online, were included in this review. However, reports about the knowledge and practice of dairy farmers towards mastitis, investigations on patterns of antimicrobial resistance of mastitis-causing bacterial infections only, and duplicate publications or extensions of the analysis from the original studies, as well as studies that were incompletely presented, were excluded from the review process. Among many of the previously published studies, only 17 met the meta-analysis selection criteria (Figure 1 and Table 1).

### 2.4. Data Extraction

The data extraction protocol consists of the name of the country, author and year of publication, study setting, sample size, number of positive cases, and prevalence of mastitis, diagnosis method used and their associated risk factors. If the study was conducted over a range of years, then the latest year of the stated range was used. The period from January 1 to March 30, 2022, was used for study selection, quality evaluation, and data extraction.

### 2.5. Quality Assessment of Individual Studies

The overall quality of the evidence was assessed using the GRADE (Grading of Recommendations Assessment, Development, and Evaluation) approach [33]. Using the three main assessment tools, the quality of each study was determined (methodological quality, comparability, and the outcome and statistical analysis of the study). High-quality publications received 5–6 points, moderate quality publications received 4 points, and low-quality articles received 0–3 points. The choice and evaluation of the articles' quality were done independently by two reviewers (AG and DT). The articles were added after agreement was reached and discrepancies between the reviewers were resolved through discussion.

### 2.6. Risk of Publication Bias

Using funnel plot symmetry, Cochran's *Q* test, and the *I*^2^ test, the risks of publication bias were analyzed. 

### 2.7. Statistical Analysis

The pooled prevalence of mastitis among dairy cows was calculated by dividing the total number of positive cases by the total number of study subjects included in this meta-analysis and multiplying by a factor of a hundred. A random-effects model was used to estimate the size of the pooled effects. To sort out the causes of heterogeneity, subgroup analysis was conducted based on sample size, region of the study, type of mastitis, and the year of publication. The Cochran *Q* statistic with inverse variance (*I*^2^) and funnel plot symmetry were used to assess the existence of statistical heterogeneity. A log odds ratio was used to determine the association between mastitis and associated risk factors among dairy cow results included in the studies. Meta-analysis was performed using Stata software version 16, where *P*≤0.05 was considered statistically significant.

## 3. Results

In Ethiopia, a total of 141 articles on the prevalence and associated risk factors of mastitis among dairy cows were recovered. Forty of these articles were excluded due to duplicates. Of the remaining 101 articles, 55 were excluded based on specific criteria included in the inclusion criteria and the data extraction protocol. Of the remaining 46 articles, 29 articles were further excluded because they did not have OR, 95% CI, and the number of positive cases (which means that the report was only based on the estimated prevalence percentage). Thus, only 17 of the studies met the eligibility criteria and were included in the final systematic review and meta-analysis study (Figure 1).

### 3.1. Causative Bacterial Agents of Mastitis

Eight eligible studies conducted in different regions of Ethiopia were purposively selected and studied the prevalence of bacterial infectionsthat cause mastitis in dairy cows. *Staphylococcus* spp. was the most prevalent bovine mastitis causative agent in dairy cows followed by coagulase-negative staphylococci (CNS), *Streptococcus* spp., *Escherichia coli*, *Bacillus* spp., *Klebsiella pneumoniae*, *Enterobacter* spp., *Corynebacterium* spp., *Micrococcus* spp., *Pseudomonas* spp., and *Arcanobacterium pyogenes* (Table 1).

### 3.2. Characteristics of the Eligible Studies

Table 2 presents the characteristics of the studies required for analysis. Seventeen studies were eligible and thus were included in the meta-analysis. Studies were conducted between 2005 and 2022, and all of them were cross-sectional studies. Eight and nine studies were carried out between 2005 and 2016 and between 2017 and 2022, respectively. Based on the criteria, four regions, namely, Benishangul-Gumuz (1 article),Amhara (3 articles), Oromia (5 articles), and SNNPR (6 articles), and the capital city, Addis Ababa (2 articles), were involved. The prevalence of bovine mastitis among eligible studies ranged between 3.9% and 73.7% (Table 2). The prevalence of clinical and subclinical mastitis in the included articles ranged from 0.9 to 48.1% and 2.5 to 56.8%, respectively (Table 2).

### 3.3. Pooled Prevalence of Bovine Mastitis

**Figure 2:**
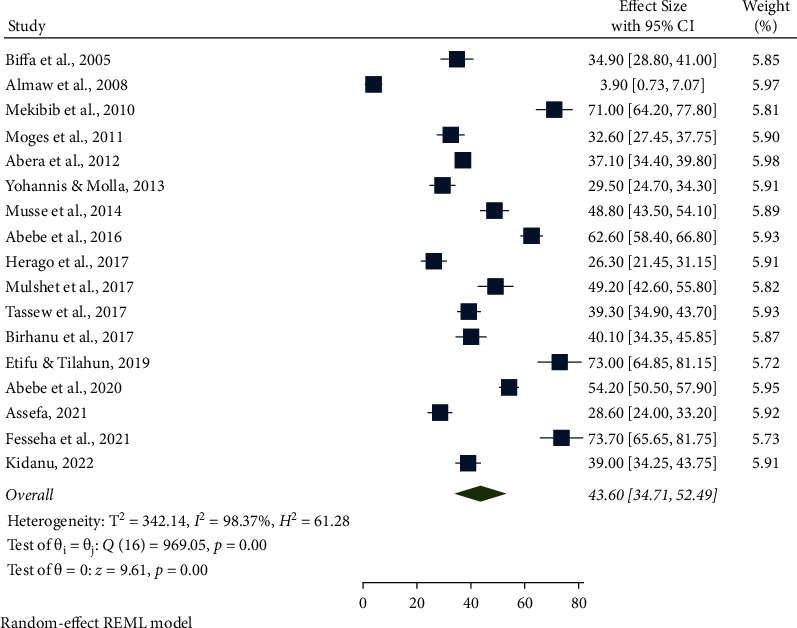
Forest plot of the pooled prevalence of bovine mastitis among dairy cows in Ethiopia from 2005 to 2022.

A random-effects model was employed to estimate the pooled prevalence of bovine mastitis among dairy cows in Ethiopia. The overall national prevalence of mastitis among dairy cows was 43.60 (95% CI: 34.71, 52.49) (Figure 2).

#### 3.3.1. Subgroup Prevalence Analysis by Region, Sample Size, Year, and Type of Mastitis

**Figure 3:**
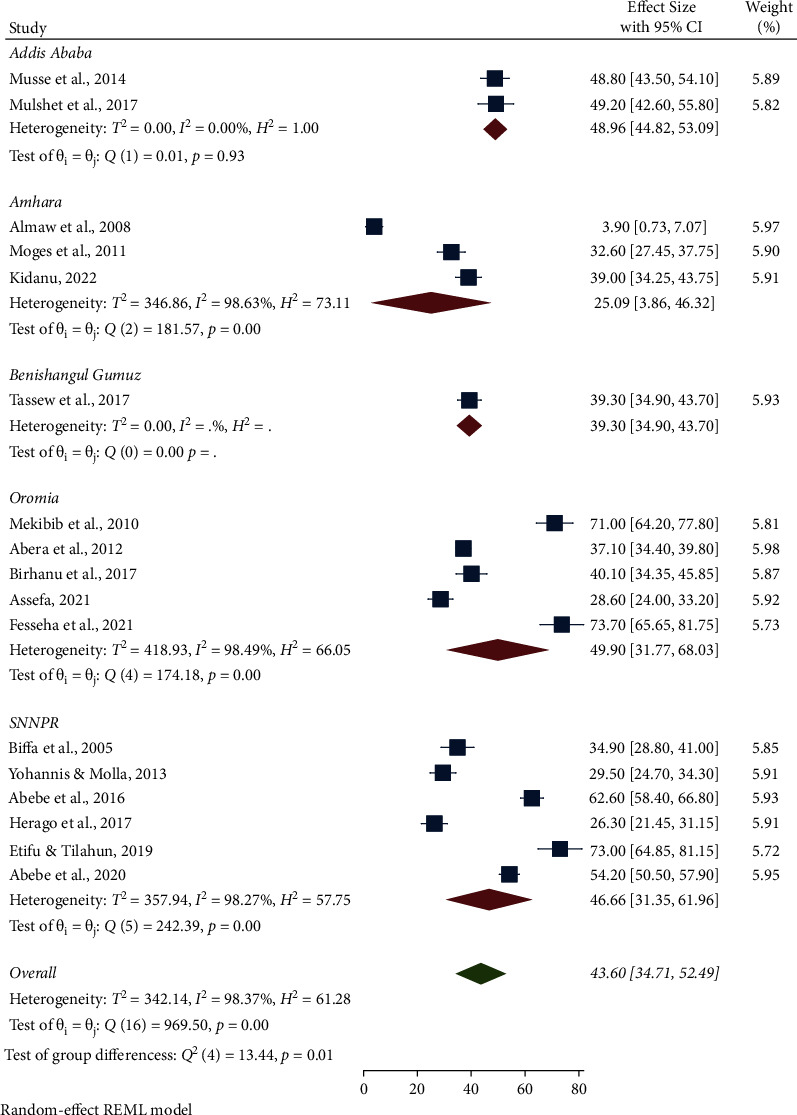
Pooled prevalence of bovine mastitis among dairy cows by region.

**Figure 4:**
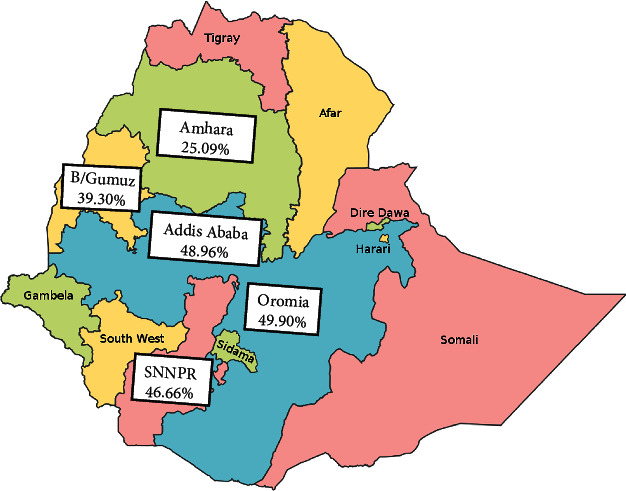
Pooled regional distribution of bovine mastitis among dairy cows in Ethiopia.

**Figure 5:**
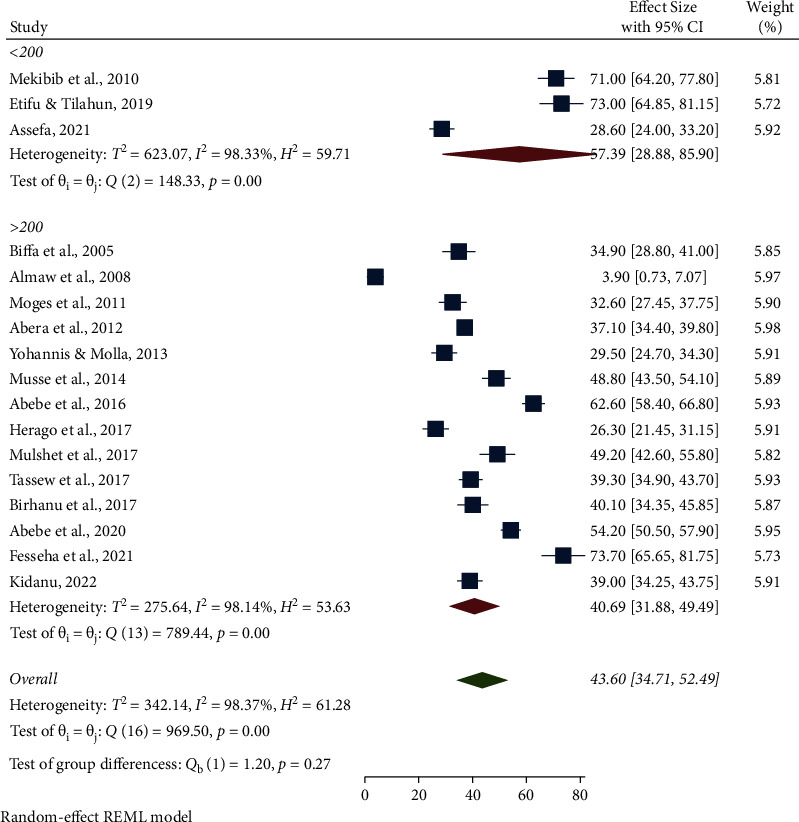
Pooled prevalence of bovine mastitis among dairy cows by sample size.

**Figure 6:**
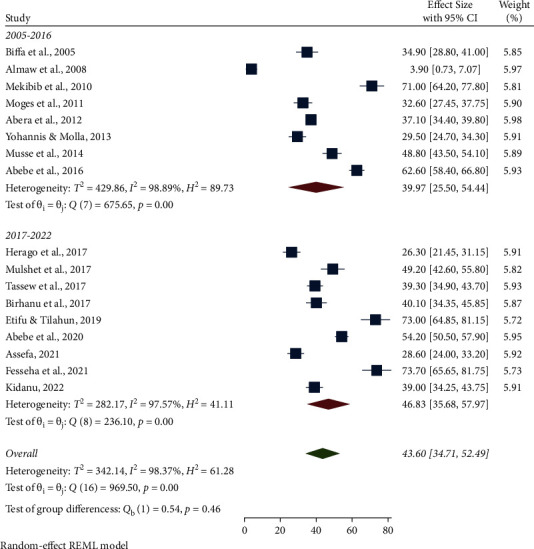
Pooled prevalence of bovine mastitis among dairy cows by year.

**Figure 7:**
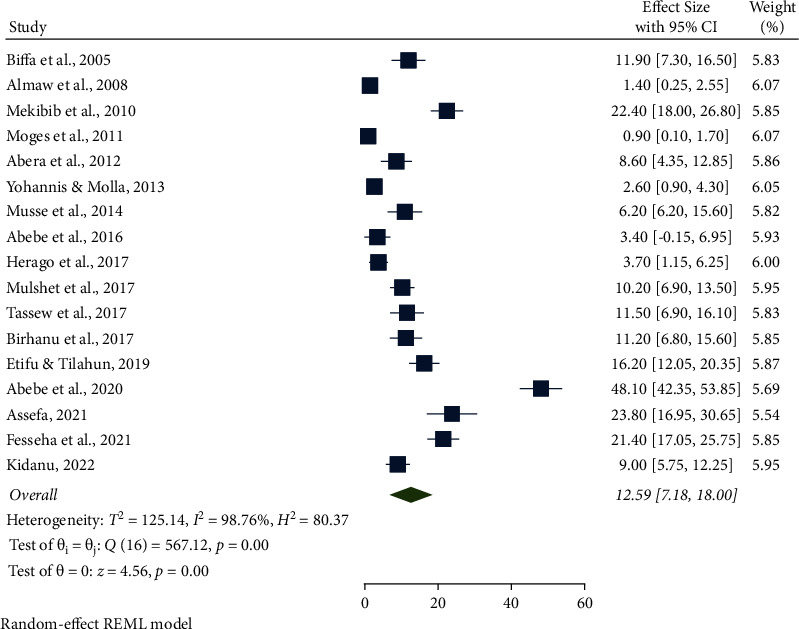
Pooled prevalence of clinical mastitis among dairy cows from 2005 to 2022.

**Figure 8:**
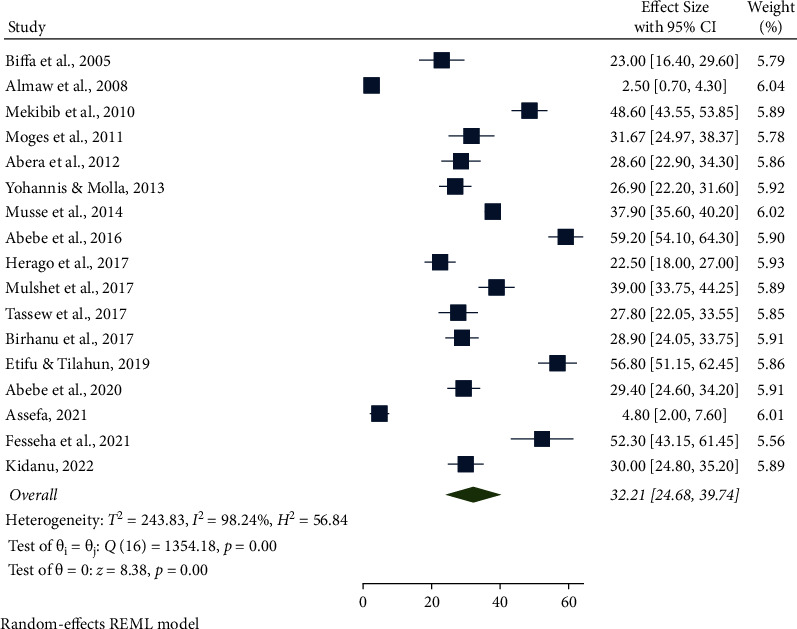
Pooled prevalence of subclinical mastitis among dairy cows from 2005 to 2022.

The highest pooled prevalence of bovine mastitis among dairy cows was reported from the Oromia region at 49.90% (95% CI: 31.77, 68.03), followed by Addis Ababa at 48.96% (95% CI: 44.82, 53.09), SNNPR region at 46.66% (95% CI: 31.35, 61.96), and Benishangul-Gumuz at 39.30% (95% CI: 34.90, 43.70), whereas a low prevalence of bovine mastitis among dairy cows was observed in the Amhara region at 25.09% (95% CI: 3.86, 46.32) (Table 3, Figures 3 and 4). The pooled prevalence of bovine mastitis among studies with sample sizes >200 (40.69%, 95% CI: 31.88–49.49) was lower than that of studies having sample sizes <200 (57.39%, 95% CI: 28.88–85.90) (Table 3 and Figure 5). The highest pooled prevalence estimate in the study period was recorded between 2017 and 2022 with a pooled prevalence estimate of 46.83% (95% CI: 35.68, 57.97), followed by the study period from 2005 to 2016 with a pooled prevalence estimate of 39.97% (95% CI: 25.50, 54.44) (Table 3 and Figure 6). The highest pooled prevalence estimate among bovine mastitis was recorded in subclinical mastitis, with a pooled prevalence estimate of 32.21% (95% CI: 24.68, 39.74), followed by clinical mastitis, with a pooled prevalence estimate of 12.59% (95% CI: 7.18, 18.00) (Table 3, Figures 7 and 8).

### 3.4. Factors Associated with Bovine Mastitis in Ethiopia

**Figure 9:**
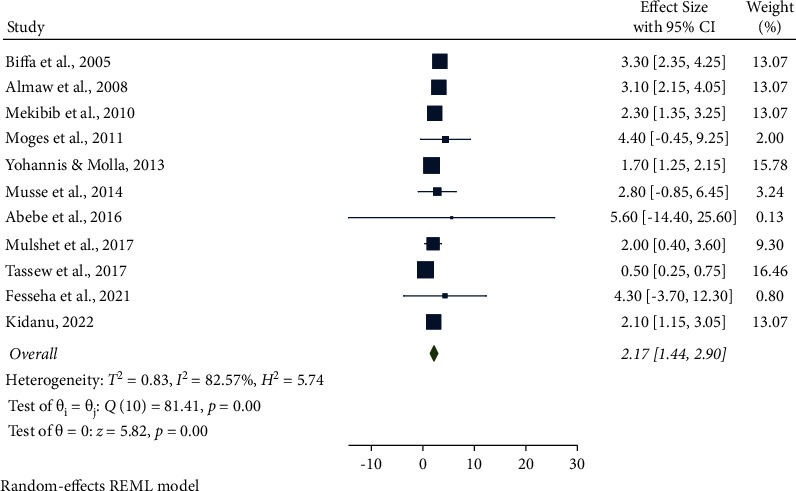
Breed as an associated risk factor for mastitis among dairy cows.

**Figure 10:**
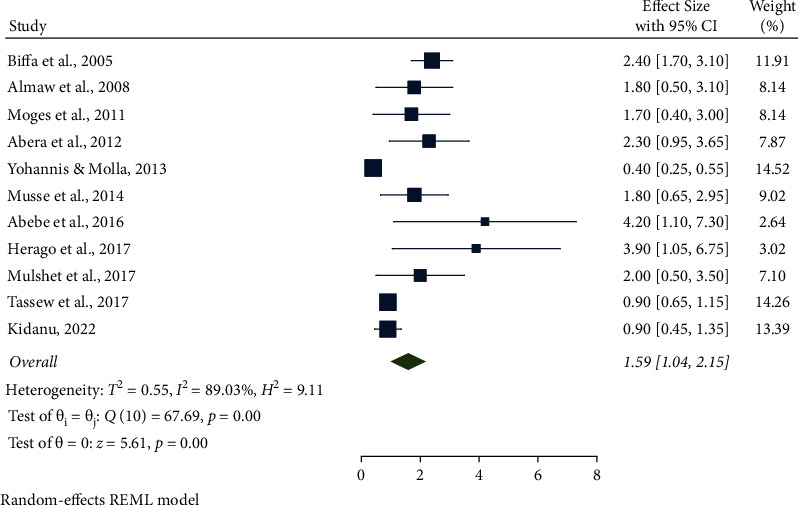
Lactation stage as an associated risk factor for mastitis among dairy cows.

**Figure 11:**
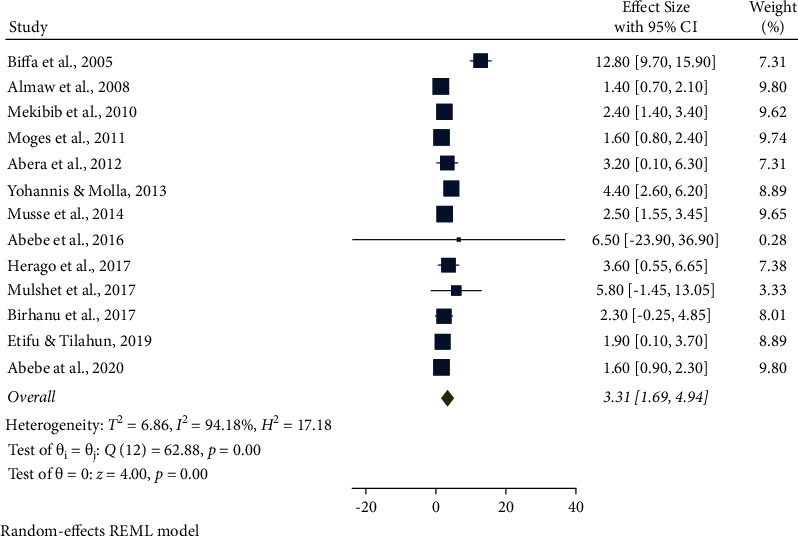
Parity as an associated risk factor for mastitis among dairy cows.

**Figure 12:**
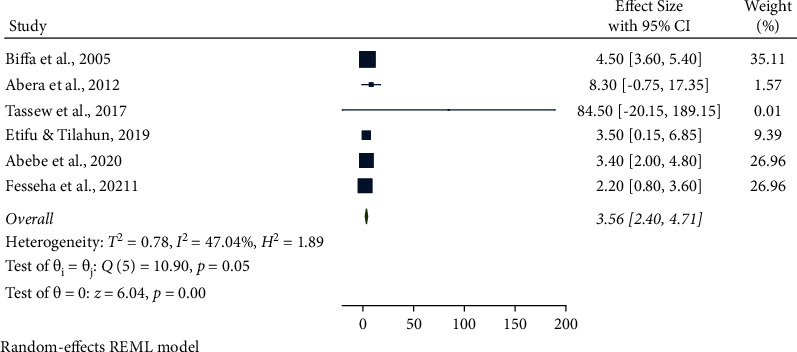
Previous history of mastitis as an associated risk factor for mastitis among dairy cows.

**Figure 13:**
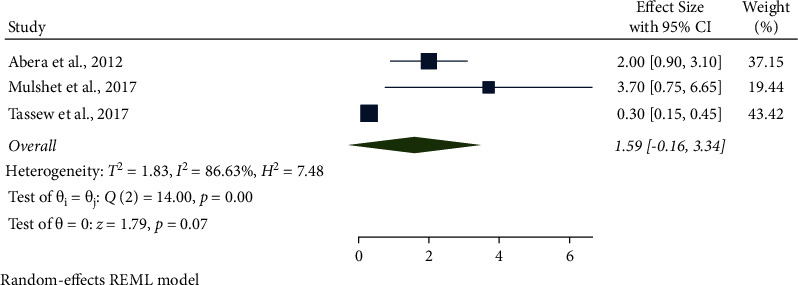
Floor type as an associated risk factor for mastitis among dairy cows.

**Figure 14:**
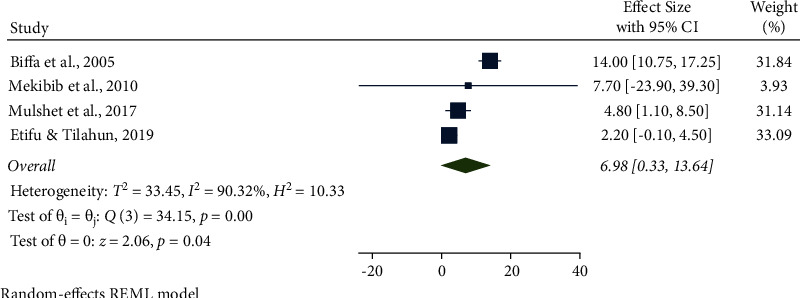
Teat injury as an associated risk factor for mastitis among dairy cows.

In this meta-analysis, several potential risk factors associated with bovine mastitis among dairy cows in Ethiopia were reviewed. However, breed, lactation stage, history of previous mastitis, floor type, and teat injury were factors significantly associated with mastitis (Figures 9to 14).

The association between breed and mastitis among dairy cows was analyzed from eleven studies. Cross-bred cows were 2.17 times (95% CI: 1.44, 2.90, *P* < 0.001) more likely to have mastitis than natives. Furthermore, the pooled result of breed was significantly associated with bovine mastitis (Figure 9).

The combined results of eleven studies showed that the lactation stage was associated with bovine mastitis. The odds of having mastitis among dairy cows were 1.59 times higher for early-stage lactation than for mid-and late lactations (95% CI: 1.04, 2.15, *P* < 0.001). Additionally, the lactation stage was significantly associated with mastitis (Figure 10).

The association between parity and mastitis among dairy cows in Ethiopia was calculated from 13 eligible studies. The AOR showing that many parities were associated with mastitis among dairy cows was 3.31 (95% CI: 1.69, 4.94, *P* < 0.001) higher than the few and moderate parities. In addition, parity as a risk factor was significantly associated with mastitis (Figure 11).

From six studies, the association between a previous history of mastitis and mastitis among dairy cows was analyzed. Cows with a history of mastitis were 3.56 times more likely (95% CI: 2.40, 4.71, *P*=0.05) to have mastitis than their counterparts. A history of mastitis was also significantly associated with the prevalence of mastitis (Figure 12).

The association between floor type and mastitis among dairy cows in Ethiopia was computed from three studies. The AOR showed that muddy soil was associated with mastitis among dairy cows and was 1.59 (95% CI: −0.16, 3.34, *P* < 0.001) higher than concrete floor types. The type of floor as a risk factor was also significantly associated with mastitis (Figure 13).

The association between teat injury and mastitis among dairy cows was analyzed in four studies. Cows with injuries to their teats were 6.98 times (95% CI: 0.33, 13.64, *P* < 0.001) more likely to have mastitis than their counterparts. Furthermore, the pooled result of the teat injury was significantly associated with the prevalence of mastitis (Figure 14).

**Figure 15:**
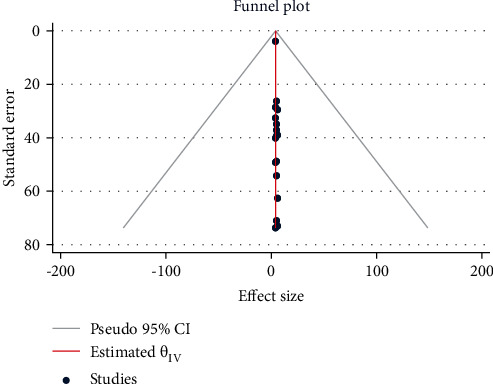
Meta funnel plot presentation, an indication of publication bias among studies in Ethiopia from 2005 to 2022.

Eleven studies (64.70%) obtained high-quality scores, while six (35.30%) had intermediate quality scores with respect to the assessment of risk of bias (Table 1). The most common biases noted were representation and case definition. The pooled prevalence of mastitis was calculated by excluding medium-quality studies to see how they affected the estimates of the overall prevalence. Pooled prevalence estimates with and without these studies had overlapped confidence intervals, indicating that there was no significant difference between them (Figure 15). Based on these findings, the majority of the primary study authors met high-quality standards (Figure 15). This gives the current findings more credibility.

## 4. Discussion

Bovine mastitis remains a major prevalent disease in cattle with a significant economic burden on the global dairy industry [43]. It causes significant financial losses on dairy farms throughout the world due to lower milk production, increased healthcare expenditures, and increased culling and death rates [44, 45]. Mastitis can also be a source of zoonosis (tuberculosis, brucellosis, and leptospirosis) and food toxin diseases (e.g., *S*. *aureus*) by allowing zoonotic transmission from bovines to humans through milk and meat, putting public health at risk [45, 46]. Antibiotics have long been seen to be the first line of defense against bacterial infections in dairy cows, particularly in the case of mastitis, when antibiotic residues can be found in the milk and microbial resistance can spread to the environment. The use of antibiotics in animal production has been researched with considerable caution due to the spread of multiple antibiotic-resistant bacteria, which is an important public health concern for animal and human health, food security, and development [47–49].

In the current study, the overall pooled prevalence of bovine mastitis among dairy cows was 43.60%. This was relatively comparable with the studies conducted in Ethiopia, SNNPR (40.40%) [7], Ambo (41.7%) [50], Holeta (44.1%) [51], Bahir Dar (44.6%) [52], Tigray (45.5%) [53], from central Ethiopian highlands (46.6%) [54], Gondar (46.9%) [55], and in a meta-analysis of Ethiopia (47.0%) [56]. The result was also similar to the findings conducted outside of Ethiopia, such as Nigeria (40.4%) [57], Brazil (40.5%) [58], Bangladesh (43.33%) [59], Somalia (44.5%) [60], and Sudan (45.8%) [61]. The result was higher than the reports in Bahir Dar (3.9%) [20], Mekelle (6.55%) [62], Addis Ababa (7.0%) [63], Sebeta (16.11%) [64], Dire Dawa (19.8%) [65], and the Sidama and Wolaita Zones (34.9%) [34] and the results outside Ethiopia, such as Nigeria (6.6%) [66], Zimbabwe (21.1%) [67], and Cameron (34.88%) [68]. However, the prevalence was lower than studies reported in Borana (59.1%) [18], Gambella (60.33%) [69], Sebeta (74.12%), Jimma (75.22%) [70], and Hawassa (76.0%) [37]. This finding is also lower than those of studies conducted outside Ethiopia, such as in Uganda (76.1%) [71], Rwanda (76.2%) [72], and Kenya (80.0%) [73]. Variation in magnitude could be due to differences in breed, study setting, study year, sample size, epidemiological status, and management system.

This study reported clinical mastitis of 12.59%. This finding was closely related to the studies conducted in Asella (10.3%) [22], Sidama and Wolaita Zones (11.9%) [34], Japan (12.0%) [74], and Hararghe (12.5%) [75]. However, the prevalence of clinical mastitis was lower than the reports of 19.6% in Addis Ababa [76], 21.1% in Borana [18], and 22.4% in Holeta [26]. On the contrary, this finding was higher than the prevalence reported as 0.93% in and around Gondar [35], 3.0% in Bahir Dar [77], 3.2% in West Shewa [78], 3.3% in China [79], 5.5% in Batu and its surroundings [80], and 6.8% in Kenya [73].

The subclinical mastitis in this study was32.21%, which is closely in agreement with the findings of 31.67% in Gondar District [35], 32.20% in Addis Ababa [81], 32.8% in Ambo District [82], 33.8% in Holeta District [83], 34.30% in and around Addis Ababa [84], and 36.67% in Sebeta District [85]. The current finding was higher than the reports of 15.2% in Gamo Zone [86], 23.10% in Wolaita Sodo [2], and 25.2% in Bahir Dar and its environs [77]. However, the subclinical mastitis recorded in this study was lower than the previous findings of 55.1% in and around Addis Ababa [76], 69.8% in Addis Ababa and its vicinity [84], 70.62% around Addis Ababa [43], 74% in Kenya [73], 76% in Hawassa [37], 76.2% in Rwanda [72], and 85% in Jimma [87]. Ineffective mastitis management strategies, environmental variables, and low hygiene standards at the sites studied could contribute to the wide variation in the prevalence of clinical and subclinical mastitis. In this finding, the incidence of subclinical mastitis was higher than that of clinical mastitis. This could be attributed to the udder's defense mechanism that limits the severity of the disease and the little attention given to subclinical mastitis while treating clinical cases. Furthermore, farmers in Ethiopia are not well informed about the silent cases of mastitis.

Regarding the bacterial causative agents of bovine mastitis, 84.70% (1015/1199) were Gram-positive bacteria (*Staphylococcus* spp., CNS, *Streptococcus* spp., *Bacillus* spp., *Corynebacterium* spp., *Micrococcus* spp., and *Arcanobacterium pyogenes*), while only 15.30% (184/1199) of the isolates were Gram-negative (*E*. *coli*, *K*. *pneumoniae*, *Enterobacter* spp., and *Pseudomonas* spp.). *Staphylococcus,* CNS, and *Streptococcus* species were the three major Gram-positive cocci commonly associated with bovine mastitis, of which *Staphylococcus* spp. constitute a major percentage (48.20%). The high prevalence of *Staphylococcus* spp. in this study is in agreement with the findings of several other researchers [18–22, 88–92]. This might be due to the hand milking and improper use of drugs. *Staphylococcus* species also cause contagious mastitis and reside predominantly within the mammary glands of cows and the skin.

In this study, the magnitude of mastitis was 2.17 times (95% CI: 1.44, 2.90) higher among cross-bred cows than among natives. Additionally, the odds of having mastitis among dairy cows were 1.59 times higher for early-stage lactation than for mid- and late lactations (95% CI: 1.04, 2.15), which is in agreement with previously reported studies elsewhere [88, 93–96]. This might be due to mastitis prevalence being influenced by various inheritable characteristics such as milk production capability, teat characteristics, and udder shape. Furthermore, it is mostly determined by the breed's genetic capability for disease resistance, the difficulty of adapting to new surroundings, and the anatomical size of the udder in cross-breeds, which is enormous and easily contaminated with bacterial pathogens. Furthermore, the prevalence of mastitis might be more common during the early stages of lactation and during the mammary gland involution period. Inconsistent results were reported elsewhere [97, 98] as when the number of lactations increased, the prevalence of mastitis increased.

The study found a strong statistical relationship between the prevalence of mastitis and the parity (AOR: 3.31 (95% CI: 1.69, 4.94)) of the animals, with the risk of mastitis increasing with parity number than few and moderate parity cows. This result was in agreement with previous studies [48–50, 56] conducted in Ethiopia. This could be due to the steady reduction in the body's immune system, anatomical changes in the udder and teats, and repetitive exposure to milking procedures, which may all contribute to the rise in the prevalence rate.

In this study, cows with a previous history of mastitis were 3.56 times (95% CI: 2.40, 4.71) more likely to have mastitis than their counterparts. A similar finding was reported in Ethiopia [99], Kenya [73], India [100], and Brazil [101]. This mastitis recurrence might be due to the insufficient screening and treatment of subclinical mastitis, as well as a lack of proper and specific identification of mastitis-causing microbial agents in clinical instances. Furthermore, farmers may indiscriminately use antibiotics, which results in the development of mastitis-resistant microbial pathogens and may also be a factor in mastitis recurrence.

In this study, cows housed on muddy soil floors were 1.59 (95% CI: −0.16, 3.34) times more affected with mastitis than those kept on a good concrete floor. Consistent findings were reported elsewhere [43, 102]. This might be due to floor contact with manure, bedding, feed, dirt, mud, and water being a potential source of mastitis-causing organisms that can easily enter the udder through the teat opening since the cow slept most of the day on it.

### 4.1. Limitations of the Study

Small numbers of published papers were collected from the regions involved in this study,and published papers from the Afar, Somali, Gambela, and Tigray regions were not included, accordingly, the prevalence of mastitis and its associated risk factors among dairy cows in Ethiopia may not be fully represented.

## 5. Conclusions

Bovine mastitis remains a major prevalent disease in cattle and places a significant economic burden on developing countries such as Ethiopia, due to the lack of problem identification and appropriate intervention measures. In the current study, the overall pooled prevalence of mastitis among dairy cows was 43.60%, of which 12.59% and 32.21% were clinical and subclinical cases, respectively. From a regional perspective, the highest and lowest pooled prevalence estimates of mastitis among dairy cows were 49.90% and 25.09% in the Oromia and Amhara regions, respectively. Gram-positive bacteria (84.70%) were the most prevalent mastitis-causing agents compared with Gram-negative bacteria (15.30%). Breed, lactation stage, history of previous mastitis, floor type, and teat injury were potential risk factors associated with mastitis among dairy cows in Ethiopia. Early detecting and treating clinical cases of mastitis in dairy cows, blanket dry cow therapy, disinfecting the teat after milking, identifying and culling chronically infected cows, and routinely maintaining milking machines are the most important control measures of bovine mastitis among dairy cows.

## Figures and Tables

**Table 1:** Total frequency and percentage of bacterial infections isolated from eight selected studies that cause bovine mastitis.

No.	Bacterial isolates	Frequency	Percentage	Included studies
1.	*Staphylococcus* species	578	48.20	Almaw et al. 2008 [20], Mekibib et al. 2010 [26], Abera et al. 2012 [27], Mulugeta and Wassie, 2013 [28], Herago et al. 2017 [29], Melesse and Minyahil, 2019 [30], Abebe et al. 2020 [31], Fesseha et al. 2021 [32]
2.	Coagulase-negative *staphylococci*	210	17.50	Almaw et al. 2008 [20], Mekibib et al. 2010 [26], Herago et al. 2017 [29], Melesse and Minyahil, 2019 [30], Fesseha et al. 2021 [32]
3.	*Streptococcus* species	153	12.76	Almaw et al. 2008 [20], Mekibib et al. 2010 [26], Abera et al. 2012 [27], Herago et al. 2017 [29], Melesse and Minyahil, 2019 [30], Abebe et al. 2020 [31], Fesseha et al. 2021[32]
4.	*Escherichia coli*	135	11.30	Mekibib et al. 2010 [26], Abera et al. 2012 [27], Mulugeta and Wassie, 2013 [28], Herago et al. 2017 [29], Melesse and Minyahil, 2019 [30], Abebe et al. 2020 [31], Fesseha et al. 2021[32]
5.	*Bacillus* species	39	3.25	Almaw et al. 2008 [20], Mekibib et al. 2010 [26], Abera et al. 2012 [27], Melesse and Minyahil, 2019 [30], Abebe et al. 2020 [31]
6.	*Klebsiella pneumoniae*	30	2.50	Mekibib et al. 2010 [26], Abera et al. 2012 [27], Melesse and Minyahil, 2019 [30]
7.	*Corynebacterium* species	19	1.58	Almaw et al. 2008 [20], Mekibib et al. 2010 [26], Mulugeta and Wassie, 2013 [28]
8.	*Enterobacter* species	16	1.33	Mekibib et al. 2010 [26], Abera et al. 2012 [27], Melesse and Minyahil, 2019 [30]
9.	*Micrococcus* species	15	1.25	Almaw et al. 2008 [20], Mekibib et al. 2010 [26], Abera et al. 2012 [27]
10.	*Pseudomonas* species	3	0.25	Abera et al. 2012 [27]
11.	*Arcanobacterium pyogenes*	1	0.08	Almaw et al. 2008 [20]

**Table 2:** List and characteristics of 17 eligible studies from 2005 to 2022.

Authors	Publication year	Region	Study design	Sample size	Case	Prevalence (95% CI)	CM prevalence (95% CI)	SCM prevalence (95% CI)	Diagnosis method used	Quality assessment
Biffa et al.	2005 [34]	SNNPR	CS	974	340	34.9 (28.7, 40.9)	11.9 (7.7, 16.9)	23.0 (13.6, 26.8)	P, B, and CMT	5
Almaw et al.	2008 [20]	Amhara	CS	351	14	3.9 (0.82, 7.17)	1.4 (0.9, 3.2)	2.5 (1.3, 4.9)	P, B, and CMT	4
Mekibib et al.	2010 [26]	Oromia	CS	107	76	71.0 (65.7, 79.3)	22.4 (18.2, 27.0)	48.6 (44.8, 55.3)	P, B, and CMT	5
Moges et al.	2011 [35]	Amhara	CS	322	105	32.6 (27.5, 37.8)	0.9 (0.2, 1.8)	31.67 (23.2, 36.6)	P, B, and CMT	4
Abera et al.	2012 [27]	Oromia	CS	422	75	37.1 (33.3, 38.7)	8.6 (4.2, 12.7)	28.6 (23.4, 34.8)	P, B, and CMT	5
Yohannis & Molla	2013 [28]	SNNPR	CS	349	103	29.5 (24.7, 34.3)	2.6 (0.9, 4.3)	26.9 (22.2, 31.6)	B and CMT	6
Musse et al.	2014 [36]	Addis Ababa	CS	346	169	48.8 (44.2, 54.8)	10.9 (6.3, 15.7)	37.9 (33.8, 38.4)	B and CMT	5
Abebe et al.	2016 [37]	SNNPR	CS	529	331	62.6 (58.3, 66.7)	3.4 (0.5, 7.6)	59.2 (54.6, 64.8)	P, B, and CMT	6
Herago et al.	2017 [29]	SNNPR	CS	320	84	26.3 (21.4, 31.1)	3.7 (1.8, 6.9)	22.5 (18.3, 27.3)	B and CMT	5
Mulshet et al.	2017 [38]	Addis Ababa	CS	390	192	49.2 (45.5, 58.7)	10.2 (7.3, 13.9)	39 (35.3, 45.8)	B and CMT	4
Tassew et al.	2017 [39]	B/Gumuz	CS	384	151	39.3 (34.7, 43.5)	11.5 (7.2, 16.4)	27.8 (21.3, 31.8)	B and CMT	5
Birhanu et al.	2017 [40]	Oromia	CS	262	105	40.1 (34.8, 46.3)	11.2 (7.4, 16.2)	28.9 (23.8, 33.5)	B and CMT	4
Etifu & Tilahun	2019 [30]	SNNPR	CS	111	81	73.0 (67.2, 83.5)	16.2 (12.5, 20.8)	56.8 (52.6, 63.9)	P, B, and CMT	6
Abebe et al.	2020 [31]	SNNPR	CS	686	372	54.2 (50.5, 57.9)	48.1 (44.3, 55.8)	29.4 (24.6, 34.2)	P, B, and CMT	5
Assefa	2021 [41]	Oromia	CS	126	36	28.6 (23.5, 32.7)	23.8 (13.7, 27.4)	4.8 (2.8, 8.4)	P, B, and CMT	4
Fesseha et al.	2021 [32]	Oromia	CS	384	283	73.7 (67.1, 83.2)	21.4 (17.5, 26.2)	52.3 (45.4, 63.7)	P, B, and CMT	4
Kidanu	2022 [42]	Amhara	CS	375	146	39.0 (33.3, 42.8)	9.0 (6.1, 12.7)	30 (25.4, 35.8)	P, B, and CMT	6

CS: cross-sectional, CM: clinical mastitis, SCM: subclinical mastitis, P: physical, B: bacteriological, CMT: California Mastitis Test, SNNPR: Southern Nations, Nationalities, and Peoples' Region.

**Table 3:** Prevalence of bovine mastitis among dairy cows in Ethiopia by subgroup analysis.

Variables	Characteristics	Number of studies	Sample size	Prevalence (95% CI)	*I* ^2^, *P* value
Sample size	<200	3	344	57.39 (95% CI: 28.88, 85.90)	98.33%, *P* < 0.001
	>200	14	6094	40.69 (95% CI: 31.88, 49.49)	98.14%, *P* < 0.001

Pooled prevalence of mastitis by region	Addis Ababa	2	736	48.96 (95% CI: 44.82, 53.09)	—
	Amhara	3	1048	25.09 (95% CI: 3.86, 46.32)	98.63%, *P* < 0.001
	B/Gumuz	1	384	39.30 (95% CI: 34.90, 43.70)	—
	Oromia	5	1301	49.90 (95% CI: 31.77, 68.03)	98.49%, *P* < 0.001
	SNNPR	6	2969	46.66 (95% CI: 31.35, 61.96)	98.27%, *P* < 0.001

Pooled prevalence of mastitis by year	2005–2016	8	3400	39.97 (95% CI: 25.50, 54.44)	98.89%, *P* < 0.001
	2017–2022	9	3038	46.83 (95% CI: 35.68, 57.97)	97.57%, *P* < 0.001

Pooled prevalence by type of mastitis	Clinical	17	6438	12.59 (95% CI: 7.18, 18.00)	98.76%, *P* < 0.001
	Subclinical	17	6438	32.21 (95% CI: 24.68, 39.74)	98.24%, *P* < 0.001
	Overall	17	6438	43.60 (95% CI: 34.71, 52.49)	98.37%, *P* < 0.001

## Data Availability

All data generated and analyzed during this study are included in this article.

## Abbreviations

AOR: Adjusted odds ratio
B: Bacteriological
CM: Clinical mastitis
CMT: California Mastitis Test
CNS: Coagulase-negative Staphylococci
CS: Cross-sectional
GRADE: Grading of Recommendations Assessment, Development, and Evaluation
P: Physical
PRISMA: Preferred Reporting Items for Systematic Reviews and Meta-Analyses
SCM: Subclinical mastitis
SNNPR: Southern Nations, Nationalities, and Peoples' Region
STATA: Statistical Software for Data Science.
